# “Not even my husband knows that I have this [breast cancer]”: survivors’ experiences in accessing, navigating and coping with treatment

**DOI:** 10.1007/s00520-024-08316-6

**Published:** 2024-01-18

**Authors:** Runcie C. W. Chidebe, Tochukwu C. Orjiakor, Gloria C. Okwu, Mary-Gloria A. Orji, Theodora O. Nwosu-Zitta, Agha A. Agha, Simeon C. Aruah, Chika Okem-Akwiwu, Candidus C. Nwakasi, Akwasi Adjei Gyimah, Zainab Shinkafi-Bagudu, Maria-Chidi C. Onyedibe, Ifeoma J. Okoye, K. Esiaka Darlingtina

**Affiliations:** 1 Project PINK BLUE—Health & Psychological Trust Centre, Abuja, Nigeria; 2 Department of Sociology & Gerontology, Miami University, Oxford, OH USA; 3 Scripps Gerontology Center, Oxford, OH USA; 4 Department of Psychology, University of Nigeria, Nsukka, Nigeria; 5 Department of Psychology, University of Toronto, Scarborough, Canada; 6 Health Policy Research Group, Department of Pharmacology and Therapeutics, College of Medicine, University of Nigeria, Enugu-Campus, Enugu Nigeria; 7 Network of People Impacted By Cancer in Nigeria (NePICiN), Abuja, Nigeria; 8 Department of Social Work, University of Nigeria, Nsukka, Nigeria; 9 Radiation Oncology Department, National Hospital Abuja, Abuja, Nigeria; 10 College of Medicine, University of Abuja, Gwagwalada, Abuja, Nigeria; 11 Breast Health Angels Foundation, Huntington Beach, CA USA; 12 Department of Human Development and Family Sciences, University of Connecticut, Storrs, CT USA; 13 Medicaid Cancer Foundation, Abuja, Nigeria; 14 College of Medicine, University of Nigeria Teaching Hospital, Enugu, Nigeria; 15 University of Nigeria Centre of Excellence for Clinical Trials, Enugu Campus, Enugu, Nigeria; 16 Center for Health Equity Transformation, University of Kentucky College of Medicine, Lexington, KY USA

**Keywords:** Breast cancer survivors, Cancer treatment, Access, Cancer experience, Cancer in Nigeria

## Abstract

**Purpose:**

Nigeria has the highest burden of breast cancer (BC) in Africa. While the survival rates for BC are over 90% in many high-income countries; low-and middle-income countries like Nigeria have 40% BC survival rates. Prior studies show that the burden and poor BC survival rates are exacerbated by both health system and individual level factors, yet there is a paucity of literature on the experiences of BC survivors in Nigeria. Hence, this study explored the divergent and convergent experiences of BC survivors in accessing, navigating, and coping with treatment.

**Methods:**

Participants (*N* = 24, aged 35 to 73 years) were recruited and engaged in focus group discussions (group 1, *n* = 11; group 2, *n* = 13 participants). Transcripts were transcribed verbatim and analyzed with inductive thematic analysis.

**Results:**

Four themes were identified: “*I am carrying this [breast cancer] alone,*” “*Living my life,*” “*‘God’ helped me,*” *and* “*A very painful journey.*” Participants described how they concealed their BC diagnosis from family and significant others while accessing and navigating BC treatment. Also, they adopted spiritual beliefs as a coping mechanism while sticking to their treatment and acknowledging the burden of BC on their well-being.

**Conclusions:**

Our findings explored the emotional burden of BC diagnosis and treatment and the willingness of the BC survivors to find meaning in their diagnosis. Treatment for BC survivors should integrate supportive care and innovative BC access tools to reduce pain and mitigate the burdens of BC.

**Implications for cancer survivors:**

The integration of innovative technologies for venous access and other treatment needs of BC is crucial and will improve survivorship. Non-disclosure of BC diagnosis is personal and complicated; hence, BC survivors need to be supported at various levels of care and treatment to make meaningful decisions. To improve survivorship, patient engagement is crucial in shared decision-making, collaboration, and active participation in care.

## Introduction

Breast cancer (BC) is the leading cause of cancer death in Nigerian women [1]. With over 28,000 new cases of BC and over 14,000 deaths in 2020, Nigeria has the highest burden of BC in Africa [2]. The incidence and death rate of BC has been increasing over time. In 2018, the Nigerian BC age-standardized incidence rate was 41.7 per 100,000 and death rates were 18.8 per 100,000 [3], compared to 49.0 per 100,000 incidence rates and 25.5 death rates in 2020 [2]. The incidence of BC in this population is projected to double by 2040, largely due to population aging and expansion [4].

While the survival rates for BC are over 90% in many high-income countries (HICs) [5], low- and middle-income countries (LMICs) like Nigeria have 40% survival rates [6] pointing to the presence of global cancer health disparity issues. BC survival rates are poor in Nigeria due to many factors including the incursion of Western lifestyle (i.e., physical inactivity and consumption of processed or unhealthy foods, alcohol, and tobacco) [7], late detection of cancer [8], poor access to cancer screening [9], poor oncology patient navigation [10], and limited access to comprehensive cancer treatment in this country [11].

Several studies have investigated the barriers and facilitators of access to BC diagnosis, treatment, survivorship, and palliative care in Nigeria. For instance, using geospatial analysis, Knapp and colleagues reported that overall, 68.9% (113 million) of Nigerians would have to travel for 4 h to access comprehensive cancer care. For a population of 206 million [12], Aruah and colleagues reported that the country requires a minimum of 280 radiotherapy machines. However, with over 124,000 cancer cases annually, Nigeria has only four of the eight government-funded radiotherapy machines that are functional at a time in the country [13]. They added that only two cancer centers can deliver advanced radiotherapy treatment and there is no positron emission tomography (PET)-CT scanner in any government cancer center in Nigeria [13]. Knapp et al. and Aruah et al. studies illustrated the lack of access to quality cancer treatment in Nigeria. Further, despite oncologic surgery being an essential treatment for 80% of cancer patients in many African countries including Nigeria, general surgeons provide this care due to the absence of specialist oncology surgeons [14]. Also, due to a lack of training and skills to deliver optimum care to cancer patients, the limited number of oncologists in centers where there is radiation and clinical oncologists are unable to adequately provide care to patients [15]. These findings suggest that the limited presence of oncological services interferes with the ability of cancer patients to experience quality cancer treatment and healthy coping after diagnoses. However, these studies examined access to cancer treatment and care from the perspective of the health system and service availability.

In a qualitative study of 15 Nigerian BC patients who underwent mastectomies, it was found that they faced various challenges in adjusting to life without a breast, including body image concerns, emotional distress, and negative societal attitudes [16]. Similarly, another study explored the experiences of 30 female cancer survivors in Nigeria and identified dualities in their access to care and support. Their findings revealed that while some survivors received compassionate care and support, others faced barriers, such as dismissive attitudes from healthcare providers, leading to feelings of abandonment and hopelessness [17]. Although the above studies provided illuminating insights into the experiences of the BC patients in Nigeria, they focused on BC survivors’ views of their life after their mastectomy and cancer survivorship, respectively.

Despite the literature described above, the experiences of BC survivors with access to treatment, coping with care, and navigation of the cancer health system in Nigeria are scarcely explored in literature. This is particularly important as the Nigerian healthcare system is weak. It will be important to isolate and describe the unique experiences of BC survivors as they try to access scarce treatment resources. To close this gap in knowledge, this present study is aimed at exploring the experiences of BC survivors in accessing, navigating, and coping with treatment and care in Nigeria and to better understand the link between healthcare and individual-level factors of coping with BC burden. On this premise, this study used the coping theory by Richard S. Lazarus and Susan Folkman. In their seminal work, coping refers to “constantly changing cognitive and behavioral efforts to manage specific external and/or internal demands that are appraised as taxing or exceeding the resources of the person” [18]. The coping theory postulates how people avoid, manage, and adapt to physical, psychological, and social harm or life-threatening situations [18]. Thus, they identified two categories of coping: emotion- and problem-focused. In emotion-focused coping, one attempts to manage internal conflicts and demands such as stressful emotions (i.e., distancing, self-control, escape-avoidance, and positive reappraisal), while problem-focused is one’s attempt to manage external conflicts and demands between the internal and the environment (i.e., seeking social support, accepting responsibility, and planful problem-solving) [18]. In addition to the categories, a taxonomy of coping explains that one’s efforts directed towards a threat are known as approach coping, while efforts that are deflected from the threat are referred to as avoidance coping [19, 20]. In connection with cancer, several studies have shown that a cancer diagnosis and its treatment have been appraised as life-threatening stressors, challenges, harm, or loss by many cancer survivors [21, 22]. Hence, survivors who appraise their cancer as a challenge are likely to use approach coping and those who appraise their cancer as harm/loss may likely use avoidance coping [23].

## Method

### Study design

This study followed an interpretive/constructivist approach as the epistemological perspective. This perspective assumes that there is no single and observable reality, rather, there are multiple realities, or interpretations, of a single event [24]. Thus, individuals develop subjective meanings of their experiences in varied and multiple ways [25]. To describe the experiences of BC patients, a qualitative descriptive approach (QDA) was chosen as the research design. QDA is a method of choice when a study is interested in straight descriptions of phenomena [26]. The perspective and design were reflective of the study’s purpose, exploring BC patients’ experiences in accessing treatment and care, describing their experiences, discovering the process through which they navigate life as BC patients, and understanding the multiple realities of living with cancer in Nigeria.

### Setting and recruitment

This study was conducted in Abuja, Nigeria, during a Breast Cancer Support Group meeting organized by a cancer nonprofit organization. The support group meeting offers cancer patients and their family members a monthly physical space to support each other, exchange knowledge, and use their shared experiences to address the challenges with cancer diagnoses, treatment, and survivorship. Although the support group was primarily for BC cancer survivors, survivors of other cancer types have joined the group. The group is currently led and moderated by BC survivors, patient navigators, and psychologists. Participants were purposively recruited from BC survivors who attended the support group meeting. Considering the aim of the study, purposive sampling gave the researchers the opportunity to include only BC survivors who possessed the characteristics of interest, that is, those who have started or completed at least one form of treatment [24]. The sampling of the cancer patients who have started or have completed cancer treatment is crucial to this study because only patients who have started or completed treatment can share their experiences with treatment. One of the authors (MAO) announced the call for participation in the study at the meeting and shared the patient information sheet with all the cancer patients and their caregivers. Participants were asked to volunteer and join any focus group of their choice. Verbal informed consent was secured from all the recruited participants. This study was performed in line with the principles of the Declaration of Helsinki, and approval was granted by the Lagos University Teaching Hospital and National Hospital Abuja Ethics Committees.

### Participants

Participants (*N* = 24) were women aged 35 to 73 years, who have been clinically diagnosed with BC. Of the 24, 11 participants volunteered to join focus group (FG) 1, and 13 joined FG 2. There was no remarkable difference between groups (i.e., FG1 and FG2). The limits of sample size in focus group research vary. Some scholars suggested six to 10 [27] and six to 12 [28] participants with a homogenous background and similar lived experiences. A smaller number of participants would allow each of them to have the opportunity to speak in detail. Based on the above, this study’s sample size was appropriate. The inclusion criteria were being a BC survivor who had started or completed any form of cancer treatment (i.e., surgery, chemotherapy, or radiotherapy). Survivors of skin cancer, new BC survivors who have not started any form of cancer treatment, and caregivers were excluded from this study. See Table 1 for the demographic and clinical characteristics of participants.

**Table 1:** Demographic and clinical characteristics of participants

Pseudonames	Age	Stages of diagnosis	Year of diagnosis	Marital status
Alban	61	III	2007	M
Bato	54	IV	2015	M
Catta	40	III	2019	S
Dahki	34	II	2019	M
Ellban	35	III	2019	S
Gibya	39	III	2019	M
Hebchi	46	III	2018	M
Idimah	35	II	2019	S
Joto	48	II	2018	M
Katwe	44	III	2019	M
Larabi	66	II	2007	W
Mafa	42	II	2019	M
Nadi	42	III	2017	M
Onasanyaa	46	0	2011	M
Petrah	46	III	2018	M
Quddux	47	I	2017	M
Rutlo	47	II	2019	M
Sachukw	73	III	2012	M
Talata	73	IV	2011	S
Utakoh	45	I	2019	M
Vivanna	44	II	2019	M
Wekpa	73	0	2010	W
Xassune	73	I	2009	W
Yata	45	III	2019	S

*M* married, *S* single, *W* widow

### Data collection

Focus group discussion (FGD) was used for the data collection in this study. FGD was the most appropriate technique considering the goal of obtaining divergence and convergence experiences of the BC survivors. In addition, FGD can be used to obtain high-quality data in a social context where participants can consider their own views and experiences in the context of the views and experiences of others [27]. An FGD guide was developed with open-ended questions based on the existing literature on experiences of living with cancer patients. Some of the questions from the guide were “*How has life been since your diagnosis?*,” “*What was your experience with cancer treatment?*,” and “*What is your experience with the cancer support group?*” During the FGD, probing questions were asked on the following topics: cancer diagnosis, cancer and work life, family and cancer diagnosis, and general well-being. The moderators were patient navigators (i.e., lay social workers) who work with Project PINK BLUE—a not-for-profit organization engaged in cancer awareness and advocacy in Nigeria. The rapport between moderators and participants was smoothly achieved as participants typically recognized them as individuals who have continued to provide support since they entered cancer care. The two FGDs were conducted on the same day in two different rooms, and they lasted for 55 min (group A) and 63 min (group B). All the FGDs were conducted in the English language and audio recorded using mobile phone recorders. After the FGD, the transcripts were manually transcribed by a research associate.

### Data analysis

Transcripts were analyzed using Braun and Clarke’s thematic analysis [29] and followed Nowell and colleagues’ steps for achieving trustworthiness [30]. Since this study used FGD with the goal of collecting diverse experiences of BC survivors with cancer treatment and care in Nigeria, thematic analysis is a useful and appropriate method for exploring the similar and different perspectives of participants and generating unanticipated insights [29, 31]. Specifically, inductive thematic analysis was used to ensure that themes generated in the study were data-driven and strongly linked to the data and not by theory or the researchers’ interest [29]. In the first step, the first author (RCWC) uploaded the transcripts to Dedoose 9.0.85, a qualitative data analysis software [32], and became familiarized with the data through prolonged engagement with the data and documentation of reflective thought. Second step, the research team used Dedoose to code all the transcripts from FGD groups A and B and general initial codes. In the third step, the researchers searched for emerging themes and engaged in triangulation. In the fourth step, the team reviewed the themes and tested for referential adequacy. Step five included defining and naming the themes, and step 6 involved the development of the analysis report.

#### Trustworthiness

For additional checks for trustworthiness and to ensure that our results are reflective of the data, we conducted a peer expert review with three leaders of the cancer support groups.

## Results

Of the 24 participants, two participants were diagnosed with stage IV BC, ten were stage III, seven were stage II, three were stage I, and two were stage 0. All the participants identified as Christians, and only one patient reported being an Atheist. A total of 17 participants were aged 30–49 years old, three were 50–69, and four were 70–73. Table 1 shows the demographic and clinical characteristics of the participants. Pseudonyms were used to protect the participants’ identities, and FG1 and FG2 were used to identify the focused group of the participants.

From the inductive thematic analysis, four themes were identified reflecting the experiences of BC survivors in accessing, navigating, and coping with treatment and care in Nigeria. All the themes were described in the participants’ own words as follows: (1) “I am carrying this [breast cancer] alone,” (2) “Living my life,” (3) “‘God’ helped me,” and (4) “A very painful journey.” Table 2 shows the themes and their sources. The themes were discussed below with supporting quotes and examples:

1. “I am carrying this [breast cancer] all alone”

**Table 2:** Themes and their sources from the inductive thematic analysis

Theme	Contributing excepts	Participants
“I am carrying this [cancer] alone”	“It’s so traumatizing. I am carrying this [cancer] all alone. With the little money I have, I cannot even ask for help because if I say it to them, honestly, they will be dead before I die.”	Mafa (FG2)
“Living my life”	“At first, it was overwhelming, but after you just have to accept, it is what it is, you just have to accept and believe that everything will change soon. So, I have been living my life.”	Dhaki (FG1)
“‘God’ helped me”	“When I came… [hospital], they said it has gone to stage III. The fear for me was the money…but God helped me, I was able to do it [cancer treatment] on my own	Onasanyaa (FG2)
“A very painful journey”	“It’s been a very painful journey, most especially when the veins collapse… I suffer a lot, maybe because I’m big… about nine pricks before I take chemo[therapy]”	Mafa (FG2)

This theme reflects participants’ perceptions of dealing with their cancer burden alone and the meaning of support in their journey for survivorship. Also, it captured how the presence and absence of support played a crucial role in the patients’ disclosure of their cancer to their families, access to treatment, and overall survivorship.

For example, Mafa (FG2) said:

It’s traumatizing for me that I lived all my life like a child… My parents kept feeding us things they said does not cause cancer because he heard that in my mom’s family, they have it [breast cancer] so he started treating it like from the onset. I haven’t been able to tell any single member of my family, not even my husband that I have this [breast cancer]. It’s so traumatizing. I am carrying this [breast cancer] all alone. With the little money I have, I cannot even ask for help because if I say it to them, honestly, they will be dead before I die.

Mafa’s comment suggests that she preferred to keep the cancer diagnosis to herself and bear the burden alone. It is unclear why Mafa believes that her family is at greater risk of dying if she discloses the diagnosis. Possibly, she believed that her family’s history of cancer is a greater concern. While Mafa shields her husband and family from knowing about her diagnosis because she believes that will put them at greater risk of dying, other participants keep their diagnosis away from their families due to stigma, fear, and lack of support. Onasanyaa (FG2) emotionally said: “I detected my own [breast cancer] early but fear didn’t allow me to come out. It was [during breastfeeding] …I just detect[ed] little seed [lump] on my breast, even to tell my husband, I did not tell him until I kept praying but the thing kept growing every day. When I came eventually [to the hospital], they said it has gone to stage III.” Onasanyaa’s description shows how participants’ experiences of “carrying [cancer] alone” propels late detection, delay in beginning cancer treatment and the possibility of poor prognosis.

Although many participants kept their cancer diagnosis and carried their cancer diagnosis alone, on the contrary, Nadi (FG2) described a divergent experience.

She said:

For me, … when I got my result, I came and dumped [dropped] it in my office…. table … and everybody started crying…my family has been very supportive, my friends, my old girls. In fact, the night I had my surgery, they [family and colleagues] did all night [prayer] till when the surgery was over. I feel so loved, like the whole world loves me, you understand. So, my family, my friends, my, in fact, every day I get calls, how are you doing, we are praying for you, so because for me the more people I told, the more prayers I got that was my own belief and you know. It has helped me a lot, it has helped me.

Just like Nadi, Dhaki (FG1) happily shared how she carried her cancer burden, she said: “The number one key to cancer treatment is love and care. So, when I was diagnosed with cancer and I told my husband… the next thing he said was, ‘You will make it, you will survive it’. So, I took it from his word. And I said that… ‘I will make it’. So, the love they gave to me, my children, my husband, and the entire family. They support[ed] me, they care for me. They always make me feel happy… they are the ones that give me hope that I am still alive.” Interestingly, participants’ experiences of bearing the burden of cancer alone or sharing with significant others differ among the participants and the kind of support systems and networks available to them. While many participants preferred to carry the cancer burden all alone and disclosed their diagnosis to only a small number of people whom they defined as significant in their lives, a few participants disclosed their diagnosis to their families, friends, colleagues, and their network which helped lessen the burden of cancer on them.

2. “Living my life”.

This theme reflects participants’ redefinition of life after their cancer diagnosis and treatment. The theme explores the positive acceptance of cancer and a new meaning of life despite their cancer diagnosis. The participants recollected the impact of cancer on their quality of life, their relationships, and work; however, they described a meaningful experience of “living” their “life.” For example, Dahki (FG1) said “At first, it was overwhelming, but after you just have to accept, it is what it is, you just have to accept and believe that everything will change soon. So, I have been living my life.” Dahki’s assertions highlight the perception of acceptance and belief while dealing with a life-threatening experience like cancer. Dahki’s view was supported by Joto (FG1)’s description.

[breast cancer] has changed my perspective of life and you know, how I see things … it has made me more patient, more understanding and made me know that you…have to take everything you see. You know, you now have to be selective about what to do, less judgmental more patient, and develop a routine so you can live a healthy life. In fact, it has made life more positive for me. More positive than otherwise.

Dahki and Joto’s descriptions of living their life after their cancer diagnosis and treatment were from personal perspectives and reflections. While Dahki speaks from the lens of hope and belief towards life, Joto speaks from the lens of self-care and a positive mindset towards life. Many of the participants reported similar positions. However, from an uncommon perspective, one of the participants took up preventive surgery after discovering that she is a carrier of breast cancer fueling-genetic mutation. Quddux (FG2) speaks in a dauntless manner mixing English and Nigerian pidgin (a local English-based creole language that is spoken as lingua franca across Nigeria), she recalled:

I had to take myself to the hospital to get my ovaries removed. So that I will stop producing estrogen… at least I am alive. I am surviving. I’m well. I didn’t have kids … I did surrogacy. I have my baby, she was a year last month… just last Sunday… I don’t care what people think… even in my office when I had my baby, they sent me on maternity leave, everybody [says] ‘we didn’t see her with pregnancy, how come?’. It’s their business o, it’s not my [business] … na me get pikin na you get your story [people who gossip, own the story, but I own the baby]. I didn’t tell you, guys, that when I was diagnosed, I was going through a divorce. But at least I am doing well, I am perfectly ok.

Quddux illustrated how she overcame the shame and stigma associated with cancer and the social situations that BC patients must navigate in their daily lives. Clearly, her decision is propelled by the goal of saving her life from cancer and living her life happily.

3. “God helped me”.

This theme reflects the religious belief and faith that “God” played a role in reducing the burden of cancer and contributed to participants’ healing. This religious belief and faith were reported by most of the participants at different levels. While so many of the participants were thankful to God for sparing their lives from cancer death, others reported that their belief, faith, and trust in God contributed to their cancer remission. Hebchi (FG1) shared her story [passionately]:

When I was first diagnosed, I was afraid. I cried. But I know that [there is] nothing God cannot do because I am a church person, I am always going to church and listening to even my pastors pray along with me, but I make sure I take my treatment and after the treatment, though I’m still feeling some pains, I know that, even inside me, I know I am healed. Medication is just formality…

Interestingly, Hebchi stressed the importance of cancer treatment amidst her religious belief regarding healing from cancer. This is not an isolated case. Just like Hebchi, Ellban (FG1) added “Ok, I take everything normal, I live a normal life because I know that with God all things are possible. So, there is nothing too big in the eyes of God. So, for that reason, I have no fear, I have been taking my treatment. By the grace of God; I have been doing well.” Larabi (FG2) added, “We spent a lot of money, borrowing money and all that. So here I am today, I have done most of my chemotherapy. I thank ‘God’ I am doing ok. I have pains here and there, but I believe ‘God’ that I am going to live out my number of years.” Hebchi, Ellban, and Larabi narrate their faith in God to bring healing for their cancer while referencing that they are taking their cancer treatment and care.

Most of the participants who referenced faith in God for healing also mentioned that they have completed their chemotherapy, radiotherapy, or have had surgery or some cancer treatment. A few others referenced God but from the perspective of securing the finance needed for their cancer treatment. Onasanyaa (FG2) reveals: “When I came… [hospital], they said it has gone to stage III. The fear for me was the money. They came out with it [cancer treatment plan], they [the doctor] said I needed nine million [naira] and … I don’t have the money and I don’t even have anybody that had that kind of money… so the fear, but God helped me, I was able to do it [cancer treatment] on my own. I spend like five million [naira] on my own until I visited you people [Project PINK BLUE] and today I thank God I am only left with… [started crying]”. Although religiosity and belief in God dominated the participants’ accounts, they acknowledged the importance of treatment and referenced having gone through one cancer treatment or the other.

4. “A very painful journey”.

This theme reflects the participants’ discomfort and challenges while trying to access cancer treatment at different levels of the healthcare system. Most of the participants elaborate that chemotherapy, radiotherapy, and other therapies are meant to treat the pain of cancer; however, they passed through severe physiological and psychological pains while trying to treat the cancerous pains. For example, Mafa (FG2) was at the clinic for a chemotherapy session and had a very “painful” experience with the insertion of a tube with a needle into the veins in her arm. She said: “It’s been a very painful journey, most especially when the veins collapse… I suffer a lot, maybe because I’m big… about nine pricks before I take chemo[therapy]. Nine full pricks! [she exclaims] Every part of my hands [are] vandalized… before I take chemo[therapy]… Because knowing that I’m going to feel pain, I’m going to feel another pain, more pain and still the result is not there [not successful]. In fact, it got to the point of using my leg and still, it was still very difficult. I would say they [doctors] are not patient… and not well trained. Their inpatient level can lead you to develop cancer on its own.”

In a similar respect, Quddux (FG2) described the issue of finding veins for her chemotherapy session as a frustrating experience. She said:

In fact, at one point, they said two days before the time I will come for chemotherapy I should be putting my hand in the freezer [to make the veins accessible]. I will be exercising it all to no avail. Sometimes I will come, they will finish, they will ask me to go, and come back two days later [because they could not locate her veins]. So, my chemo[therapy] was never steady because I will always go and come back. After five years [of treatment], I was waiting for a clean bill of health. Another one [breast lump] came out here, I went for a biopsy [laboratory test] they say it’s cancerous [recurrence]. I started another round of chemo[therapy]. That was two years ago, that was really what got me pissed. After two chemo[therapy sessions], the whole thing [problem] of getting my vein, I just told them, I will not do chemo[therapy] again.

While Mafa and Quddux shared their painful journey from the lenses of finding veins, Petrah (FG2) and Nadi’s (FG2) experiences were deeply rooted in poor access. Petrah gave a compelling account, she said: “It was very traumatic because the doctors were arguing amongst themselves whether it’s cancer or not. A doctor in a [public] hospital told me it’s nothing. So, I now went to a private hospital and got another biopsy [surgical removal of body tissue for laboratory test] and they sent the sample to the public hospital. But the public hospital was on strike and the sample was left there. So, by the time they now resumed… the thing [cancer] has now spread. Immediately, I went in for chemo. I did four courses and then surgery. After which I did another four and then went for radiotherapy…I have a lot of stress because I have an invalid husband. Some days he will die and resurrect, so the stress was too much on me.” Petrah’s account draws attention to the effect of social support in helping BC patients cope with treatment and care. The lack of help from her husband, whom she equates to a dead person, appears to worsen both her physical and psychological well-being.

Some participants described challenges of navigating the complex cancer healthcare system and how poor access to timely diagnosis and treatment were contributors to their painful journey with cancer. Nadi tearfully recalled how her delayed lab result from a public hospital forced her to seek care and treatment at a private hospital. She said:

Well, when I notice the lump, I just stopped breastfeeding. I did the biopsy, and it came out positive [cancerous]. And I told my husband let’s get a second opinion but when I did that after the biopsy, it took one whole month for the result to come out at the public hospital. My husband had to, even go, shout, and knock on their door. That pulled me off, and I don’t want to go back to the public hospital, and I now fell into the hands of the private hospitals where… for every chemotherapy I took, I paid 35,000 [Naira]. The doctor wanted to do surgery and it failed, that was when they now invited an oncologist. You see why it was wrong for me to go to a private hospital because there was no oncologist … The oncologist said I should start chemotherapy first.

Nadi’s experiences in navigating the complex cancer healthcare system by going from public to private cancer center were also narrated by many other participants. Further, Nadi explained that access to advanced diagnostic equipment for cancer detection is an issue even in public hospitals. She added:

When I started chemotherapy, I couldn’t do a bone scan because there was no chemistry [reagents] …for the test and so I had a spread [of cancer] in my bone. And if there was also a PET (positron emission tomography) scan [in Nigeria] … all these would not have happened. Maybe we would have done that [PET scan] and then know the spread of cancer and how to start treatment. Today, I have gone back to the public hospital and that is the only place where I can get radiotherapy. In fact, the doctor that did my surgery came from the United Kingdom. He was saying he sends all his patients to South Africa and Ghana.

**Fig. 1:**
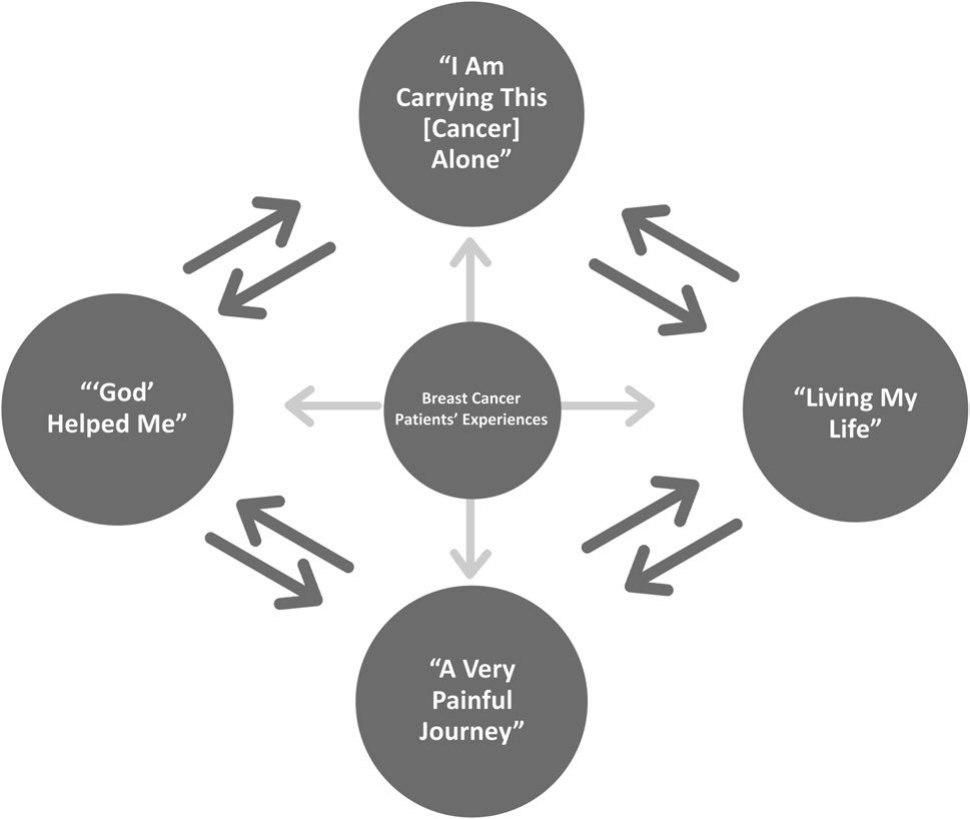
Thematic connections of breast cancer patients’ experiences

In summary, all the participants’ experiences with care and treatment reflect the extent to which a weak healthcare system, poor cancer treatment equipment, and poor oncology workforce can impact their well-being and survivorship, particularly what they view as a painful journey with cancer. Some of the participants have accepted their fate and are living their life, while others use their religious belief, that is “God helped” them. The participants’ accounts show that the nature of relationships, family support, and social networks are meaningful to how they “carried” their cancer burden. The themes are interconnected in diverse ways: “I am carrying this [cancer] alone” led many participants to accept the disease and began “Living [their] life”. The weak healthcare system and poor support conveyed by patients as “a very painful journey” has led to “‘God’ helped me,” for solace and support. These experiences showcase pockets of support that help women in low-resource regions buoy through the deficiencies in the local health system. A deeper understanding of these supportive beliefs and structures and working to leverage them may hold the potential to better the lot of people with BC in low-resource regions. See Fig. 1 for the thematic connections.

## Discussion

Experiences of BC survivors in accessing, navigating, and coping with treatment and care in Nigeria and many LMICs have been a subject of interest in cancer survivorship studies and patient engagement discussions. Nonetheless, very few studies have explicitly explored these issues. On this premise, the goal of this study was to explore divergent and convergent experiences of BC survivors in accessing, navigating, and coping with treatment and care in Nigeria. Our hope is that these experiences narrated will help in better understanding of their difficulties and areas of potential strengths that can be explored to improve BC survivorship.

### Non-disclosure of BC diagnosis, access to treatment, and family support

The main findings of this study show that concealing and non-disclosure of BC diagnosis and bearing the burden alone is a contributing factor to delayed access to BC treatments, poor prognosis, and poor BC survivorship. Some of the participants hid their diagnoses from their significant others, children, friends, and families. This may have impacted their ability to utilize problem-focused coping in navigating the cancer treatment and care [18], thus impacts their capacity to engage in approach coping, seeking social support and information on how best to navigate, access, and cope with the limited BC treatment facilities. This non-disclosure and concealing of BC diagnosis by the participants point to the importance of understanding sociocultural contexts in addressing the burden of BC in Nigeria. Nigerian cancer control is marred by disparities in cancer prevention [33], weak health insurance [34], lack of needed equipment [13], and limited skills and personnel [15]. Hence, non-disclosure of BC diagnosis may further make it challenging for survivors to have access to limited BC treatment. Previous studies have shown that even when there is access to cancer healthcare services, some individuals can experience significant delays in cancer treatment [35, 36]. Thus, Nigeria’s National Cancer Control Plan (2023–2027) and the National Institute for Cancer Research and Treatment should recognize how the healthcare system level, survivors’ preferences, and sociocultural factors interact to worsen the burden of BC among survivors. The oncology multi-disciplinary team (MDT) should be ready and willing to have open and honest discussions about the situation of cancer treatment in Nigeria- admitting what is available, feasible, and possible. These discussions should always involve and accommodate the experiences of BC survivors and those who support them, admitting the healthcare inadequacies as well as the strengths that families and communities provide to fill in the gaps for system inefficiencies. These discussions can help bolster community education and awareness about cancer and prepare people to support cancer control advocacy campaigns as well as make them ready to support survivors in their communities and immediate families.

In addition, this study found that participants who disclosed their diagnosis to families and social networks received support in navigating, accessing, and coping with their BC treatment. This finding is in line with previous studies that reported that family support is important to cancer survivors since family is the closest system and plays a crucial role in coping strategy [37, 38]. For participants, leveraging problem-focused coping strategies, managing external conflicts, and seeking and obtaining support from family and friends may improve their mental well-being and BC survivorship [18]. Similarly, participants who accepted their BC diagnosis and defined BC as a challenge are most likely to use approach coping and possibly have a better quality of life (QoL). Given the current findings and the role of family support as it reverberated across many narratives in the data, it is important that oncological treatment and care consider the inclusion of supportive family members very early in the treatment process while respecting patients’ decisions to disclose or not to disclose. Clinicians should inquire from BC patients early in the treatment process to consider including family members and maybe significant others who could be supportive in the treatment process. It is not uncommon to experience resistance as patients often feel that they are protecting family members from the pain and worry of their conditions. The current research however provides a reference for practitioners and service users, to the experiences of similar patients who found family and significant others to be productively supportive. This is very important, especially in low-resource regions where healthcare benefits and welfare programs are scarce, and most health expenditures are funded out of pocket. There are other hidden family support systems and networks that are common, especially in Africa. These resources could also be explored to gather as much support as possible for patients. Family systems are typically quick to mobilize support for kith and kin when they are seriously ill and could quickly exempt them from social/family/financial obligations if they understand the severity of health issues. Communities within which these family systems are nested could also benefit from education programs that make them aware of the nature of the cancer disease, the health system gaps, and the need for community structure to mobilize support for people with such diagnoses. While the family and community systems are important, patient engagement through peer navigation, peer support groups, and advocacy training are important. For instance, BC survivors could be trained on how to provide peer navigation to newly diagnosed women and create awareness and policy advocacy for improved care in different states of the country.

### Access to BC treatment

Another key finding is the participants’ report on their challenging experiences with the pains of finding veins for chemotherapy, delay getting radio diagnostics results, absence of PET CT scanners in public facilities, limited bone scans, poor clinical oncology workforce, and other health system challenges. For instance, many participants reported that the difficulty in finding veins (i.e., venous access) for the administration of chemotherapy was a very painful experience. In Nigeria, chemotherapy is mostly administered using intravenous (IV) catheters—the process of having a doctor or nurse put a needle in the forearm or part of the body to deliver treatments directly to the veins of the patients. While IV treatments are beneficial to BC survivors, the process of inserting the IV treatment (i.e., venipuncture) can be discomforting and painful for patients who require recurrent treatments for a longer time [39]. On this premise, totally implantable venous access ports (TIVAPs) otherwise known as chemo ports used to deliver prolonged therapies [40]. With the insertion of TIVAPs in BC patients, frequent needle pricks are eliminated, less frequent flushing, reduced risk of infection, less interference with daily activities, and more comfortable [40–42]. In cases where TIVAPs are not available, vein-viewing devices have been found to be useful in decreasing the time and number of needle pricks [43]. However, the use of TIVAPs or vein-viewing devices is not widespread in Nigeria and many LMICs due to a lack of surgical skills for implantation, cost, and availability [44]. The implication of this finding is that there is an urgent need for clinicians to explore TIVAPs and give BC survivors options. The government and global cancer control communities need to also consider investing in training surgeons to have the skills and embrace innovative technologies that improve the quality of life of BC survivors. On the other hand, poor access to bone scans and the absence of PET CT scanners in public facilities are huge challenges, especially for a country where about 98% of women present advanced breast cancer at diagnosis [8]; these services are regularly needed but not always available. There is an urgent need to scale up cancer treatment infrastructure in this population.

### Religiosity and BC

Further, religion or belief in the supernatural was found to be a vital component of how participants cope with BC treatment and care. The finding points to how emotion-focused coping strategies are leveraged by the participants to manage internal conflicts for positive reappraisal and self-control [18]. Many respondents referred to religious phenomena as a core place of strength, a strength that is critical for dealing with the physical and psychological burden of BC. Interestingly, this present study also showed how religiosity interacts with coping, and access to BC treatment with two surprising findings. First, many participants stressed the importance of BC treatment while holding their strong religious beliefs. This finding is in contrast with previous findings that showed that BC survivors refused treatment due to their religious beliefs [17]. Second, some participants referenced religiosity from the perspective of securing financial support for their BC treatment. The implication of this finding is that there is a need for healthcare leaders and clinicians to begin to think of the benefits of religious belief and bodies beyond individual coping. Religious bodies could be used to secure financial support for the treatment of indigent BC survivors and as an avenue for social support. Africans and particularly Nigerians hold their religious beliefs and practices in high regard [45]. As such, BC awareness and education could target places and circles of worship to raise awareness and build support networks for BC survivors. Such a strategy has been found to be very useful for delivering health programs. For example, a congregation-based setting was used to improve HIV testing and the use of antiretroviral therapy among pregnant women [46]. Similar approaches could be deployed in cancer control, especially to raise awareness for cancer screening, early detection, and treatment access. Finally, Project PINK BLUE’s model of a psychological support center for cancer patients could be replicated in treatment facilities and among support groups to ameliorate the mental challenges that arise as a result of cancer treatment and care [47].

## Strength, limitations, and future directions

The use of FGD was a strength of this study which explored divergent and convergent data. Using inductive thematic analysis was also appropriate as it raised the voices of the patients in their own words and context. Despite the important contributions of the current study, it is not without limitations. The participants were recruited in the cosmopolitan capital city of Abuja. The experiences of participants may portray the lives of people living in urban metropolises and not the experiences of people living in rural parts of Nigeria. Abuja metropolis also has a functional publicly funded oncology care center. Accessing treatment may in fact be more difficult for people in rural areas. It is important to note that the current participants are part of a cancer support group. Given the shared social support and resources available to them, their experience of life after cancer would likely not mirror the experiences of people that are not part of any cancer support group. Additionally, there was a wide variation among participants regarding the time from diagnosis to the time of the study. This may mean that short-term and long-term experiences living with the condition are muddled up. However, both long- and short-term experiences are valid, and we do not think that they influenced the lessons learned from the experiences. The findings reflect the experiences of BC survivors and may not be generalizable to survivors of other types of cancer. Future studies should explore the experiences of survivors with other cancer types in comparison with BC survivors.

## Conclusion

The burden of BC is devastating and increasing in Nigeria. BC survivors in Nigeria are faced with navigating both the limited treatment facilities that contribute to poor prognosis and coping with personal challenges that arise from being diagnosed with BC. While some patients intentionally conceal their diagnosis as a strategy for navigating BC treatment and care outside the scrutiny of family and friends, others accept their diagnosis and factor it into their lifestyles and relationships with others (i.e., families, co-workers, and friends). Better BC healthcare systems are needed to improve BC-related outcomes for Nigerians. Family and community support systems should also be strengthened to assist BC patients in navigating their care plans. Nigeria’s new National Cancer Control Plan should be integrated into the federal budget to ensure it is funded for improved BC cancer control in Nigeria.
